# The *Col4a2^em1(IMPC)Wtsi^* mouse line: lessons from the Deciphering the Mechanisms of Developmental Disorders program

**DOI:** 10.1242/bio.042895

**Published:** 2019-07-22

**Authors:** Lukas F. Reissig, Anna Nele Herdina, Julia Rose, Barbara Maurer-Gesek, Jenna L. Lane, Fabrice Prin, Robert Wilson, Emily Hardman, Antonella Galli, Catherine Tudor, Elizabeth Tuck, Cecilia Icoresi-Mazzeo, Jacqueline K. White, Ed Ryder, Diane Gleeson, David J. Adams, Stefan H. Geyer, Timothy J. Mohun, Wolfgang J. Weninger

**Affiliations:** 1 Division of Anatomy, MIC, Medical University of Vienna, Waehringer Str. 13, 1090 Vienna, Austria; 2 The Francis Crick Institute, 1 Midland Road, London NW1 1AT, UK; 3 Wellcome Trust Sanger Institute, Wellcome Genome Campus, Cambridge CB10 1SA, UK

**Keywords:** Developmental disorders, Standardised phenotyping, Collagen, HREM, DMDD, Rare diseases

## Abstract

The Deciphering the Mechanisms of Developmental Disorders (DMDD) program uses a systematic and standardised approach to characterise the phenotype of embryos stemming from mouse lines, which produce embryonically lethal offspring. Our study aims to provide detailed phenotype descriptions of homozygous *Col4a2^em1(IMPC)Wtsi^* mutants produced in DMDD and harvested at embryonic day 14.5. This shall provide new information on the role *Col4a2* plays in organogenesis and demonstrate the capacity of the DMDD database for identifying models for researching inherited disorders. The DMDD *Col4a2^em1(IMPC)Wtsi^* mutants survived organogenesis and thus revealed the full spectrum of organs and tissues, the development of which depends on *Col4a2* encoded proteins. They showed defects in the brain, cranial nerves, visual system, lungs, endocrine glands, skeleton, subepithelial tissues and mild to severe cardiovascular malformations. Together, this makes the DMDD *Col4a2^em1(IMPC)Wtsi^* line a useful model for identifying the spectrum of defects and for researching the mechanisms underlying autosomal dominant porencephaly 2 (OMIM # 614483), a rare human disease. Thus we demonstrate the general capacity of the DMDD approach and webpage as a valuable source for identifying mouse models for rare diseases.

## Abstract (precis)

**Summary:** We define the spectrum of phenotypic abnormalities linked with *Col4a2* disruption and demonstrate the opportunities the Deciphering the Mechanisms of Developmental Disorders (DMDD) program offers for exploring rare human diseases.

## INTRODUCTION

The Deciphering the Mechanisms of Developmental Disorders (DMDD) program is an international, systematic effort to identify and characterise the phenotypes of mouse embryos carrying embryonic or perinatal lethal mutations (Dickinson et al., 2016; Geyer et al., 2017c; Mohun et al., 2013; Weninger et al., 2014; Wilson et al., 2016). Comprehensive phenotype data of the embryos of 208 knockout lines are available online (https://dmdd.org.uk/), enabling individual researchers to identify lines relevant to their research.

The heart of DMDD is the characterisation of the phenotype of embryos harvested at embryonic day (E) 14.5. At this developmental stage, organogenesis is complete and comprehensive analysis of the role gene products play in organ formation is possible, even if defects resulting from gene mutation cause death in the foetal or perinatal period (Geyer et al., 2017b; Wilson et al., 2017).

In the DMDD pipeline, a *Col4a2* mutant line [*Col4a2^em1(IMPC)Wtsi^*] was generated. Together with *Col4a1*, *Col4a2* encodes extracellular matrix proteins, which participate in the macromolecular network of basement membranes (Hudson et al., 1993; Jeanne and Gould, 2017; Khoshnoodi et al., 2008; Sado et al., 1998). Basement membranes are thin, amorphous, specialised extracellular matrices, which function as an anchor for epithelial cells (Pozzi et al., 2017). Hence, they are essential components of all vessels and many organs and play a vital role in diverse biological events including embryogenesis, tissue remodelling, wound healing, protection of tissues and organs from exogenous factors, resistance to mechanical stress, and filtration of blood and air (Sado et al., 1998).

Consistent with the distribution of basement membranes and *COL4A2* products, mutations of *COL4A2* have been found to cause severe disorders with abnormalities of the nervous, vascular and visual system in humans (Jeanne and Gould, 2017; Meuwissen et al., 2015; Verbeek et al., 2012). Abnormalities of other organs are not described yet, except for the occurrence of a renal cyst associated with hematuria in a patient with heterozygote *COL4A2* deletion (Gunda et al., 2014). However, systematic studies failed to detect *COL4A2* mutations in families with hematuria and symptoms of thin basement membrane nephropathy (TBMN) (Zhang et al., 2007).

Autosomal dominant porencephaly 2 (OMIM #614483), a recently described and rarely diagnosed disease, can be caused either by segment deletions of chromosome 13, which carries the *COL4A1* and *COL4A2* genes, or by mutations of one of the *COL4A2* alleles (Huang et al., 2012; Kirchhoff et al., 2009; McMahon et al., 2015; Meuwissen et al., 2015; Quélin et al., 2009; Vahedi et al., 2007; Yoneda et al., 2012). Heterozygous carriers of *COL4A2* mutations chiefly suffer from symptoms caused by brain and eye defects. Brain abnormalities include cavities in the cerebral parenchyma, which result from developmental defects (Verbeek et al., 2012) or recurrent haemorrhagic strokes due to germinal matrix haemorrhage or bleeding from blood vessels with defective basement membranes (Meuwissen et al., 2015; Murray et al., 2014).

In contrast to patients with *COL4A2* mutations, patients with *COL4A1* mutations show ­­– in addition to brain and cardiovascular malformations – defects of the kidneys and muscles (Jeanne and Gould, 2017; Meuwissen et al., 2015). Patients with segment deletions of chromosome 13, including deletion of both *COL4A1* and *COL4A2* as well as other genes, also suffer from a range of abnormalities, including brain, heart, lung, kidney and muscle defects, but also craniofacial and skeletal abnormalities (Huang et al., 2012; McMahon et al., 2015; Yang et al., 2013).

In the 1980s, first attempts were made to use the mouse as a model for researching the function of *COL4A2*, with studies mainly focusing on eye phenotypes (Favor, 1983; Favor, 1986; Favor and Neuhäuser-Klaus, 2000). In those, and a study in which embryos with point mutations were produced from random mutagenesis experiments [*C3.Cg-Col4a2^ENU415^/Ieg*, *C3.D2(Cg)-Col4a2^ENU4003^/Ieg*, *C3.D2(Cg)-Col4a2^ENU4020^/Ieg*], most homozygous embryos died around mid-gestation (E10.5–11.5). Only five *Col4a2^ENU415^* embryos survived organogenesis and were still alive at E15. These showed developmental delay, microphthalmia and extensive haemorrhages. In contrast, heterozygous mice survived the postnatal period, but suffered from eye defects, cutaneous haemorrhage and cerebral abnormalities (Favor et al., 2007). Lung malformations were found in heterozygous *C3.D2(Cg)-Col4a2^ENU4003^/Ieg* embryos at E18.5 (Loscertales et al., 2016). Strikingly, despite the important role of COL4A2 in the formation of basement membranes ubiquitously distributed in the mammal body, no defects in any other organ systems were identified in mouse embryos lacking both *Col4a2* alleles.

In the scope of the DMDD program, we produced a *Col4a2* mutant line, harvested homozygous mouse embryos at E14.5 and generated high-resolution digital volume data of these embryos with the high-resolution episcopic microscopy (HREM) technique. This study aims to present detailed descriptions of the morphological phenotype of the *Col4a2* mutants and examine the implications of the DMDD project in identifying and researching models of rare diseases by using autosomal dominant porencephaly type 2 as an example.

## RESULTS

Comparisons of the three wild-type littermates collected from the *Col4a2^em1(IMPC)Wtsi^* mutant strain with a reference collection of stage-matched controls (204 embryos in total) on the same genetic background did not reveal notable malformations. For the *Col4a2^−/−^* mutants however, such comparisons revealed severe abnormalities in almost all organs (Table 1) and a delay in the development and growth of the individuals. Using the staging system of Geyer et al., complemented by crown-rump-length and Theiler staging in embryos in which the limbs were malformed (Geyer et al., 2017b), showed that all individuals appeared younger than expected for the time of harvesting.

**Table 1.**
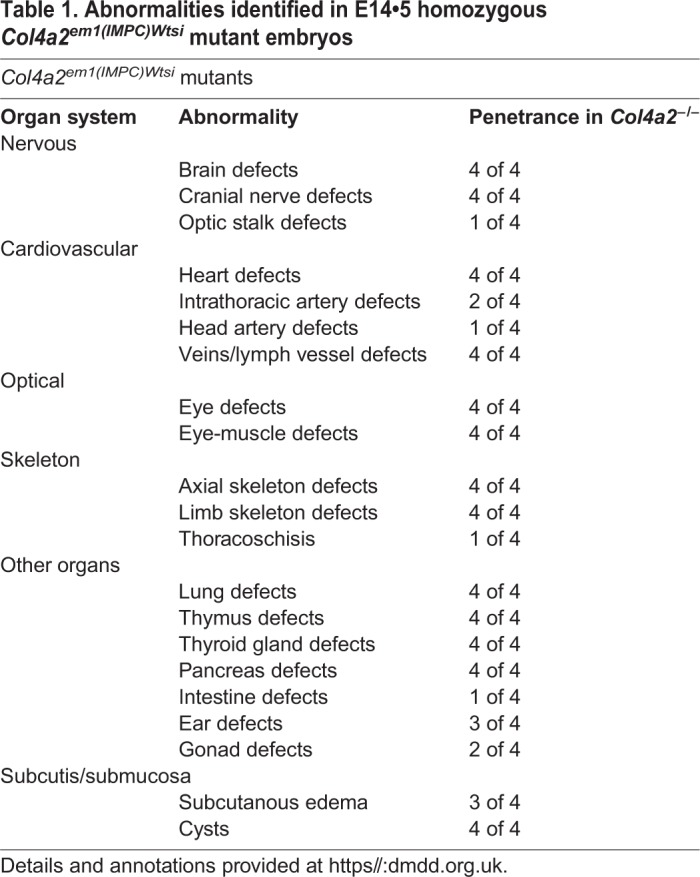
**Abnormalities identified in E14•5 homozygous *Col4a2^em1(IMPC)Wtsi^* mutant embryos**

All *Col4a2^−/−^* mutants had a large number of defects of almost all organs, with the most complex malformations affecting the nervous, cardiovascular and skeletal systems.

### Nervous system

**Fig. 1.**
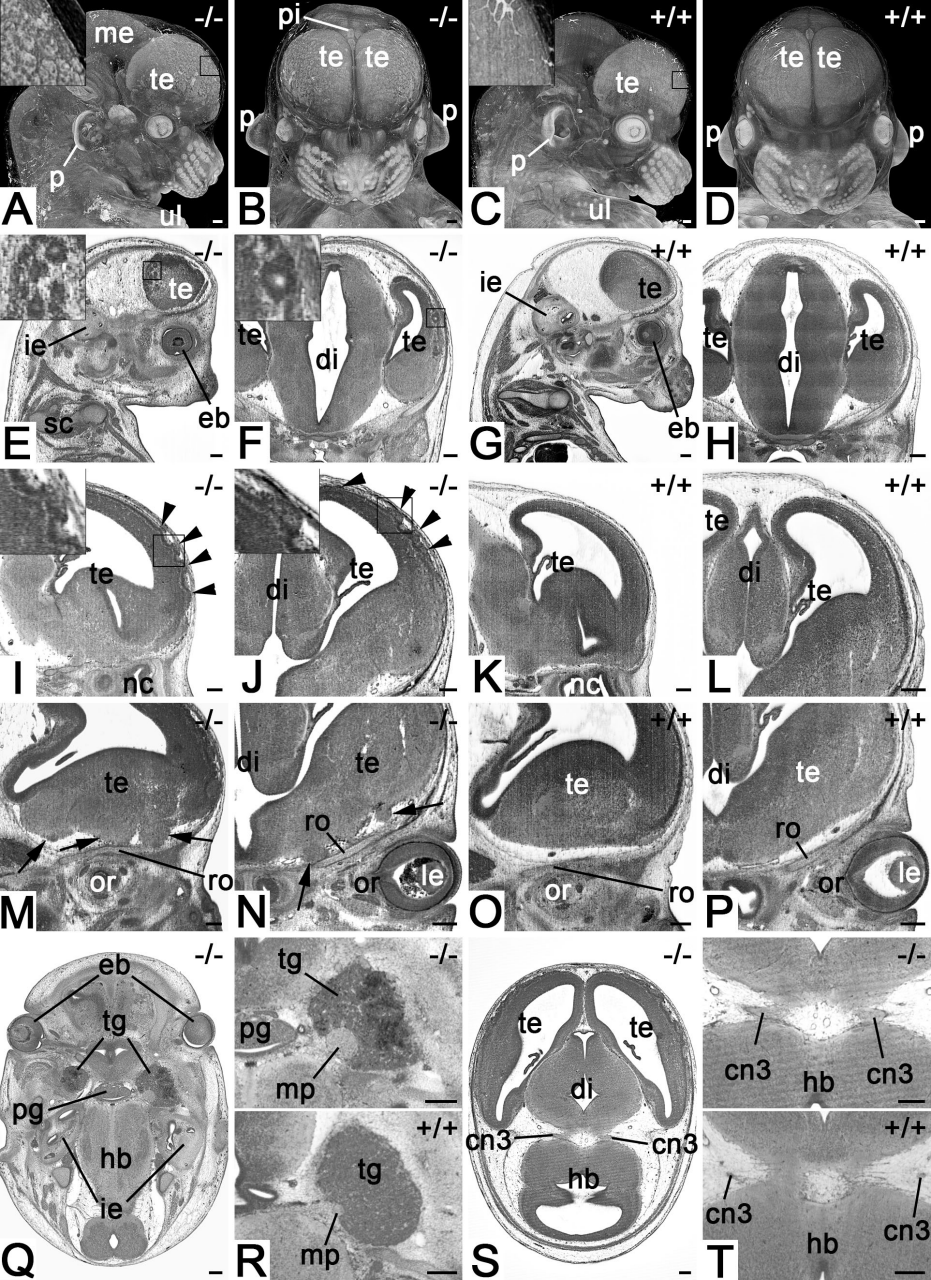
**Defects of the nervous system in *Col4a2^em1(IMPC)Wtsi^* mutant mice.** (A,B) Appearance of the forebrain in semitransparent volume models of head and neck. View from lateral (A) and ventral (B). Note the irregular structure of the surface depicted in the inset. (C,D) Controls. (E,F) Cystic structures in the cortex of telencephalon. Sagittal (E) (ventral to the right) and coronal (F) resections through HREM data. (G,H) Controls. (I,J) Surface irregularities (arrowheads) of the superolateral cortex of telencephalon. Sagittal (I) (ventral to the right) and coronal (J) resection through HREM data. (K,L) Controls. (M,N) Tissue protrusions (arrows) at the basal forebrain. Sagittal (M) (ventral to the right) and coronal (O) resection through HREM data. (O,P) Controls. (Q,R) Thickening of motoric portion of trigeminal nerve and haemorrhage in the trigeminal ganglion. Axial sections from cranial. Ventral on top. Top panel of R magnifies ganglion and nerve. Bottom panel serves as control. (S,T) Abnormal oculomotor nerve. Axial sections from cranial. Ventral on top. Top panel of T magnifies roots of the oculomotor nerve. Bottom panel serves as control. Abbreviations: cn3, oculomotor nerve; di, diencephalon; eb, eyeball; hb, hindbrain; ie, inner ear; le, lens; me, mesencephalon; mp, motoric portion of trigeminal nerve; nc, nasal cavity; or, orbit; p, pinna; pg, pituitary gland; pi, pineal gland; ro, roof of orbit; te, telencephalon; tg, trigeminal ganglion; ul, upper limb. Scale bars: 200 µm.

All four mutants (100%) showed brain and cranial nerve abnormalities (see Movies 1–3). The most intriguing features were large numbers of very small tissue irregularities and tissue protrusion on the superolateral cerebral cortex resulting in a ‘bumpy’ appearance of the surface (Fig. 1A,B,I,J), and multiple small cyst-like structures beneath the lateral surfaces of the left and right telencephalic hemispheres (Fig. 1E,F). In addition, the basal face of the frontal lobe showed multiple solid and irregularly shaped, but voluminous tissue protrusions displacing the leptomeningeal connective tissues and olfactory fibres (Fig. 1M,N). The motoric portions of the trigeminal nerves were enlarged and haemorrhages were detected inside the trigeminal ganglia (Fig. 1Q,R). The oculomotor nerves had fewer but thicker roots and larger diameters than in controls (Fig. 1S,T).

### Cardiovascular system

**Fig. 2.**
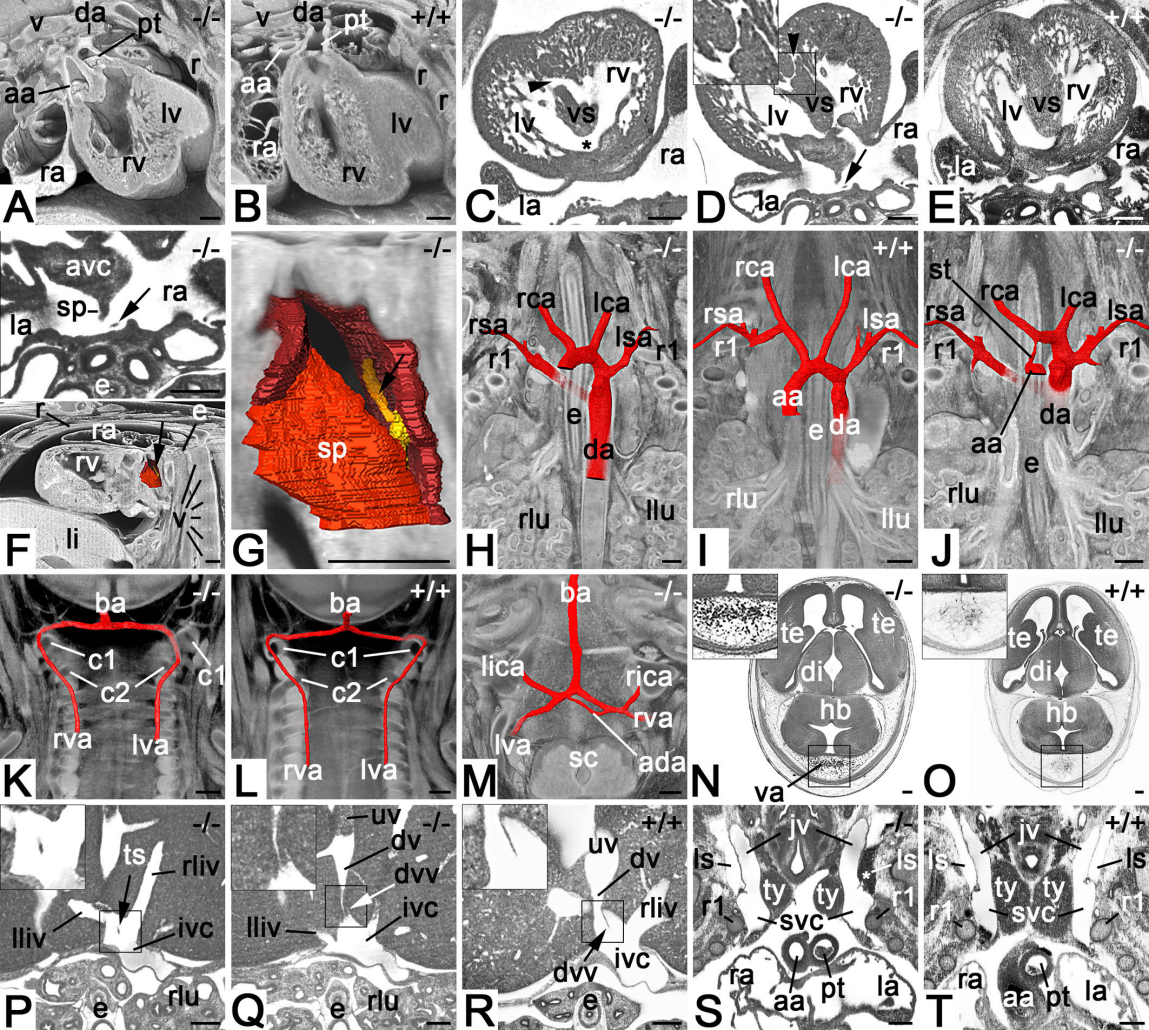
**Cardiovascular malformations in *Col4a2^em1(IMPC)Wtsi^* mutant mice.** (A) Double-outlet right ventricle. Axially sectioned volume models from cranio-ventral. Dorsal on top. (B) Control. (C) Combined perimembranous (asterisk) and large muscular (arrowhead) ventricular septal defects. Axial HREM section from cranial. Ventral on top. (D) Small muscular ventricular septal defect (arrowhead). Axial HREM section from cranial. Ventral on top. Note the tissue strand (arrow) in the dorsal atrium. (E) Control. (F) Abnormal tissue strand (arrow) connecting septum secundum and dorsal atrium wall. Surface models of dorsal atrium, septum primum and septum secundum (all in red) together with a sagittally and axially sectioned volume model of the thorax from lateral. Ventral to the left. Top panel (magnification of D) shows a section through the tissue strand. (G) Magnified surface model of F. Note the model of the tissue strand (yellow, arrow). (H–J) Malformations of the great intrathoracic arteries. Surface models of arteries (red) combined with coronally sectioned volume models of the cranial thorax from ventral featuring a retroesophageal right subclavian artery (H), normal situs (I) and combined stenosis of ascending aorta and retroesophageal right subclavian artery (J). (K) Abnormal topology of left vertebral artery. Surface models of vertebral and basilar arteries combined with coronally sectioned volume model of the neck from ventral. Note that the left vertebral artery enters the spinal canal below the arcus of the atlas instead of above. (L) Control. (M) Additional anastomosis between intracranial segments of left and right vertebral arteries. Surface models of arteries combined with an axially sectioned volume model of the spino-cranial junction. (N) Abnormal intracranial blood vessels dorsal to the hindbrain. Axial HREM section. Ventral on top. (O) Control. (P–R) Axial HREM sections through cranial liver segments and inferior vena cava. Tissue strand crossing junction of liver veins and vena cava inferior (P). Abnormal valve of ductus venosus (Q). Control (R). (S) Blood in left lymph sac (asterisk). Coronal resection through HREM data showing the cranial thorax and neck from ventral. Note the distance between the thymus lobes. (T) Control. Abbreviations: aa, ascending aorta; ada, additional anastomosis; avc, atriovetricular cushion; ba, basilar artery; c1, arcus of atlas; c2, arcus of axis; da, descending aorta; di; diencephalon; dv, ductus venosus; dvv, valve of ductus venosus; e, esophagus; hb, hindbrain; ivc, inferior vena cava; jv, jugular vein; la, left atrium; lca, left common carotid artery; li, liver; lica, left inferior cerebellar artery; lliv, vein from left liver lobe; llu, left lung; ls, lymph sac; lsa, left subclavian artery; lv, left ventricle; lva, left vertebral artery; pt, pulmonary trunk; r, rib; ra, right atrium; rca, right common carotid artery; rica, right inferior celebellar artery; rliv, vein from right liver lobe; rlu, righ lung; rsa, right subclavian artery; rv, right ventricle; rva, right vertebral artery; sc, spinal chord; sp, septum primum; st, aortic stenosis; svc, superior vena cava; te, telencephalon; ts, tissue strand; ty, thymus; uv, umbilical vein; v, vertebra; va, abnormal blood vessels. Scale bars: 200 µm.

All mutants (100%) showed a broad spectrum of complex cardiovascular defects. This included serious heart malformations such as double-outlet right ventricle (Fig. 2A), atrioventricular cushion abnormalities, muscular ventricular septal defect (Fig. 2C,D), bicuspid aortic valve and atrial septal abnormalities (Fig. 2F,G), but also abnormalities of the venous system, especially abnormal morphology or absence of the valve of the ductus venosus (Fig. 2Q).

In two of the mutants, these abnormalities were associated with malformations of the great intrathoracic arteries, such as right retroesophageal subclavian artery (Fig. 2H,J) and stenosis of the ascending aorta (Fig. 2J).

Furthermore, each of the mutants showed at least one of the following phenotypes (penetrance in parenthesis): abnormalities of the vertebral artery (1/4) (Fig. 2K,M), the capillaries (3/4) (Fig. 2N), the inferior vena cava (1/4) (Fig. 2P), the hepatic veins (1/4), and of the connections between the subcutaneous lymph vessels and lymph sacs (2/4) (Fig. 2S).

### Skeleton

All mutants (100%) had defects of the axial and limb skeleton.

**Fig. 3.**
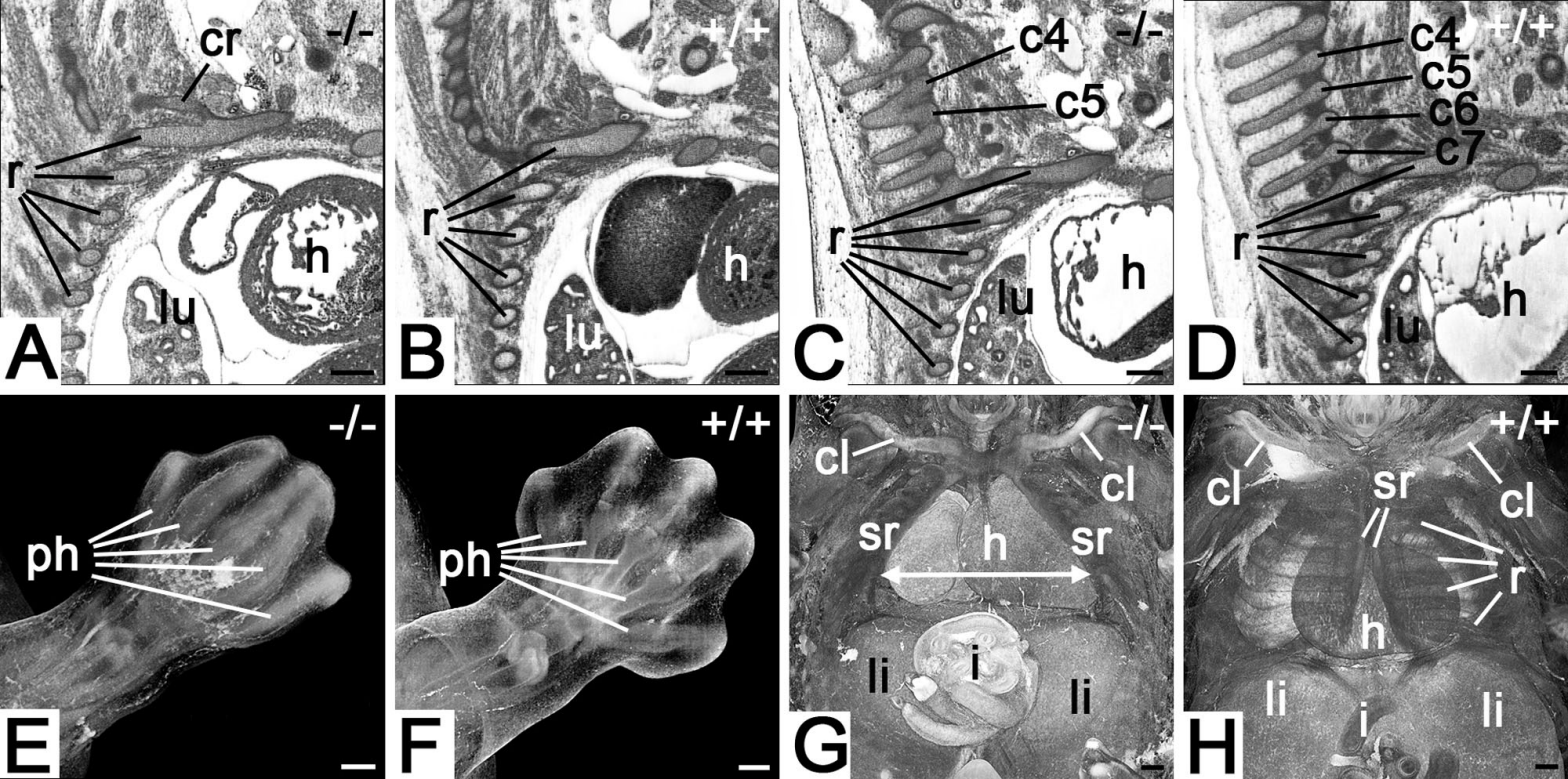
**Skeletal abnormalities *Col4a2^em1(IMPC)Wtsi^*** **mutant mice.** (A) Abnormal cervical rib connected to 1st rib. Sagittal resection through HREM data. Ventral to the right. (B) Control. (C) Fusion of vertebral arches of 4th and 5th cervical vertebrae. Sagittal resection through HREM data. Ventral to the right. (D) Control. (E) Malformed foot plate. Volume model from dorsal. Note the syndactyly and the abnormal patterning of the phalanges. (F) Control. (G) Thoracoschisis (arrow). Volume model of thorax from ventral. Note the distance between the sternal ridges (arrow). (H) Control. Abbreviations: c4, 4th cervical vertebra; c5, 5th cervical vertebra; c6, 6th cervical vertebra; c7, 7th cervical vertebra; cl, cavicle; cr, cervical rib; h, heart; i, intestine; li, liver; lu, lung; ph, phalanges; r, ribs; sr, sternal ridge. Scale bars: 200 µm.

In the axial skeleton, all components were affected to various degrees, with all mutants (100%) showing rib abnormalities (Fig. 3A), such as abnormal cervical ribs, fused ribs, absent costovertebral joints and vertebra abnormalities, such as fused vertebral arches (Fig. 3C).

In the limb skeleton, abnormalities of the distal parts of the limbs were identified in all four (100%) of the mutants (Fig. 3E).

Thoracoschisis (Fig. 3G) occurred in one mutant.

### Other organs

**Fig. 4.**
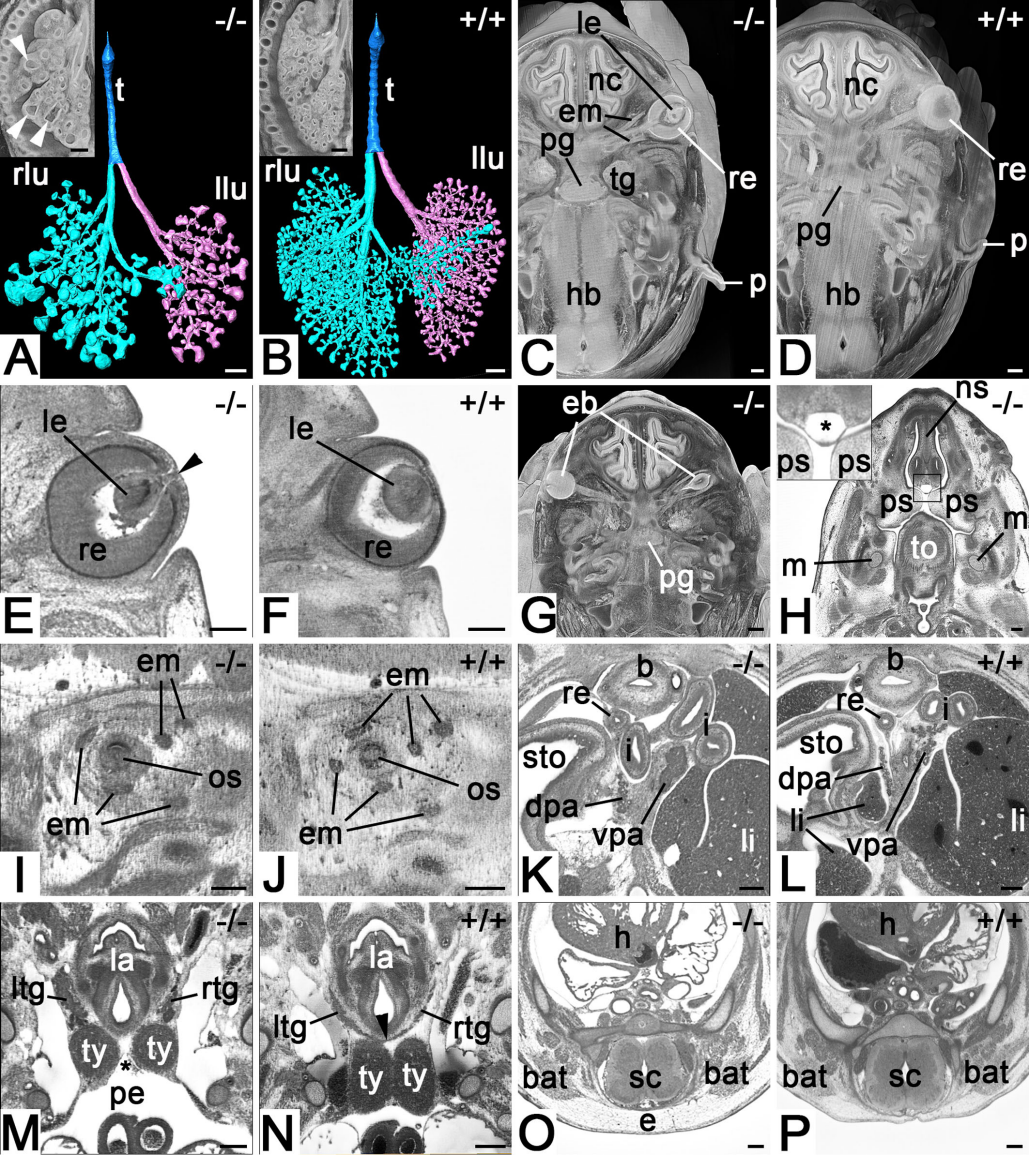
**Defects of lung, eye, ear and endocrine organs in *Col4a2^em1(IMPC)Wtsi^* mutant mice.** (A) Abnormal branching and enlargement of terminal airways (arrowhead). Surface model of trachea, bronchi and alveoles from ventral. Coronal section through a volume model of the right thorax and lung in the inset. Note the growth delay. (B) Control. (C) Abnormal pinna and right lens. Axial section through the head of a volume model from cranial. Ventral on top. (D) Control. (E) Connection of lens with overlying epidermis (arrowhead) indicating delayed eye development. Axial HREM section. (F) Control. (G) Right eyeball inside the head mesenchyme. Axial section through the head of a volume model from cranial. Ventral on top. Compare with D. (H) Subepithelial cyst (asterisk in inset). Axial HREM section from cranial. Ventral on top. (I) Missing eye muscle. Sagittal resection through HREM data of the orbit. Ventral to the right. Note that only five of six eye muscles are present. (J) Control. (K) Abnormal ventral pancreas. Axial HREM section from cranial. Ventral on top. Note the solid tissue of the ventral pancreas. (L) Control. (M) Missing isthmus of thyroid gland (arrowhead in N) and abnormal size and position of left and right thymus. Coronal resection through HREM data from ventral. Note the extension of the pericardial cavity between both thymus lobes (asterisk). (N) Control. (O) Subcutaneous edema. (P) Control. Abbreviations: b, urinary bladder; bat, brown adipose tissue; dpa, doral pancreas; e, subcutaneous edema; eb, eyeball; em, eye muscle; h, heart; hb, hindbrain; i, intestine; la, larynx; le, lens; li, liver; llu, left lung; ltg, left lobe of thyroid gland; lu, lung; m, Meckel's cartilage; nc, nasal cavity; ns, nasal septum; os, optic stalk; p, pinna; pe, pericardial cavity; pg, pituitary gland; ps, palatine shelf; re, rectum; rlu, right lung; rtg, right lobe of thyroid gland; sc, spinal cord; sto, stomach; t, trachea; tg, trigeminal ganglion; to, tongue; ty, thymus; vpa, ventral pancreas. Scale bars: 200 µm.

In addition to the defects in the skeleton and nervous and cardiovascular system, all mutants (100%) showed abnormalities of their lungs (cystic enlargements of their terminal airways; Fig. 4A), thymus, thyroid glands, and pancreas (Fig. 4K,M) as well as an unusual, thin-walled cystic structure, which occupied quite a large space in the mesenchymal tissue along the midline between nasal septum and oral cavity (Fig. 4H).

Additionally, each of the mutants showed at least one of the following abnormalities (penetrance in parenthesis): a clearly discernible subcutaneous edema (3/4) (Fig. 4O), outer ear abnormalities (3/4) (Fig. 4C), eye or eye-muscle defects (3/4) (Fig. 4E,G,I), abnormal testis morphology (1/4), abnormal placement of parts of the intestine (1/4) and a small accumulation of tissue in the thoracic cavity (1/4).

### Gene expression analysis

Gene expression analysis of three homozygous *Col4a2^em1(IMPC)Wtsi^* embryos harvested at E9.5 showed some residual *Col4a2* gene expression (an 11× knockdown; 1/0.084) using a TaqMan assay 3′ to the deletion exon. *Col4a1* showed wild-type expression levels. For detailed results, see Supplementary Information (Table S1).

## DISCUSSION

Patients with single-allele mutations of *COL4A2* suffer from autosomal dominant porencephaly type 2, cerebral small vessel disease, recurrent intracerebral haemorrhages (ICH), hydrocephalus, schizencephaly or severe eye defects. The chief symptoms of porencephaly type 2 are periventricular cystic lesions, hemiplegia, developmental delays, intellectual disability, microphthalmia, myopia and optic nerve hypoplasia.

Other types of porencephaly show a similar spectrum, but are caused by mutations of *COL4A1* or segment deletions of chromosome 13, which affect both the *COL4A2* and *COL4A1* genes. For researching the role *COL4A2* plays in normal development and in the genesis of porencephaly, knockout mouse models were engineered (Favor, 1983; Pöschl et al., 2004) and it quickly became evident that null mutants are prenatally lethal. Unfortunately, in most cases the homozygous individuals died before they had the chance to complete embryogenesis (Favor, 1983; Favor, 1986; Pöschl et al., 2004). This effectively prevented a comprehensive characterisation of the function of the COL4A2 products by analysing structural defects due to *Col4a2* deletion.

In contrast, the DMDD program created a *Col4a2^em1(IMPC)Wtsi^* mutant line, which produces homozygous individuals that survive organogenesis. In order to seek an explanation for this, we conducted gene expression analysis of three homozygous *Col4a2^em1(IMPC)Wtsi^* embryos harvested at E9.5. These studies showed some residual *Col4a2* gene expression (an 11× knockdown; 1/0.084), which most likely explains the longer survival times of homozygous embryos produced from the *Col4a2^em1(IMPC^^)Wtsi^* line.

The advantage of this residual activity is that the DMDD line overcomes the limitations of most of the earlier models and permits identification of a large spectrum of important structural defects caused by *Col4a2* deletion. This recommends the DMDD strain for studies aiming at researching the function of COL4A2 products in normal development and for developing new procedures for researching, diagnosing and treating autosomal dominant porencephaly type 2.

All examined *Col4a2* mutant embryos (100%) have severe tissue abnormalities in the superficial layers of the cerebral cortex. Some lesions are cystic, others solid. This endorses the interpretation that Col4a2 products are essential for the formation of the membrana limitans gliae superficialis and its extension, the membrana limitans gliae perivascularis (Favor et al., 2007). We think that these membranes fail to properly organise the target area for migrating neural cells and, as a result, the migration and settlement of neural cells during cortex development is severely disturbed. Translated to humans, such abnormal cortical arrangement might cause symptoms such as reduced intellectual and motor abilities and epilepsy, which are indeed described to be associated with autosomal dominant porencephaly type 2 (Ha et al., 2016; Jeanne and Gould, 2017; McGovern et al., 2017; Meuwissen et al., 2015; Verbeek et al., 2012).

So far, an abnormal cortical tissue arrangement as observed in mice is not described in humans. However, it is highly likely that this might have escaped diagnosis, since the defects are small enough to go unnoticed in routine diagnostic procedures. In a comparable manner, this might as well be true for the abnormalities of the cranial nerves we observed in mice, but which were not yet described in humans. Building on our mouse data, we hypothesise that patients suffering from autosomal dominant porencephaly type 2 will benefit from checking for the existence of such small defects with the aid of specialised imaging techniques.

All the examined mouse mutants showed heart defects of a severity that is likely to explain prenatal death. But interestingly, the spectrum of the heart defects exceeded the spectrum expected from the distribution of gene expression data. According to earlier studies *Col4a2* activity occurs in the atrioventricular valves, the great intrathoracic vessels, and the epicardium (Hanson et al., 2013; Klewer et al., 1998; Sado et al., 1998). Thus, it was unexpected that 100% of the mutants we examined had muscular septal defects. The absence of *Col4A2* activity in the ventricular septum led us to the conclusion that the muscular septum defect is highly likely to result from abnormal biomechanical forces in the region of the muscular part of the septum, which are caused by the abnormally formed atrioventricular junction. This is an interesting hypothesis in itself, but also makes the *Col4a2* mutant strain a promising model for researching the influence of biomechanical forces on cardiovascular development and remodelling.

*COL4A2* products play an important role in the formation and function of all basement membranes (Sado et al., 1998). Such membranes are not restricted to the brain or heart, but exist in many organs throughout the body (Kuo et al., 2012). Consistent with this, we were able to show that in mouse embryos with deletion of both *Col4a2* alleles, almost all organs are affected, but neither heart malformations nor malformations of the skeleton and many major organs have been described in previous gene ablation studies. One reason for this might be the early lethality of the homozygous embryos, which concealed organ defects and the differences in the manner of phenotyping (Favor et al., 2007; Kuo et al., 2014; Pöschl et al., 2004). Another might be that our phenotyping approach was based on 3D high-resolution digital HREM data analysed according to the standardised protocol and the guidelines developed for the DMDD program (Mohun et al., 2013; Weninger et al., 2014). However, we cannot rule out that the genetic background on which the mutations were studied has a significant influence on the penetrance and severity of pathologies.

Basement membranes also play a major role in airway development (Warburton et al., 1999) and are components of the skin and exocrine and endocrine glands (Sado et al., 1998). This explains why our *Col4a2* mutant embryos show an abnormal branching pattern of the lower airways with reduced numbers of oversized alveoles and feature subcutaneous edema and cysts as well as defects of the pancreas and thyroid gland. The observed defects of the endocrine organs are structural, but they might result in functional defects as well. Whether this can be translated to humans is not clear. Nevertheless, it might be beneficial to test patients suffering from porencephaly for endocrine disorders, such as diabetes and hypothyroidism.

Defective function of either *Col4a1* or *Col4a2* products or deletion of both genes cause similar defects (Jeanne and Gould, 2017; Meuwissen et al., 2015). Furthermore, the *Col4a1* and *Col4a2* genes are located on the same segment of chromosome 13 and share a promoter region. Hence, we checked *Col4a1* expression in three homozygous *Col4a2^em1(IMPC)Wtsi^* embryos harvested at E9.5 in order to exclude a possible disruption of the gene in the *Col4a2^em1(IMPC)Wtsi^* line created in the DMDD program. Since all examined embryos showed wild-type expression levels of *Col4a1*, we feel it safe to consider the phenotype abnormalities we observed to be a direct result of *Col4a2* disruption*.*

Our study is based on phenotype information of only four embryos. Nevertheless, most features occurred in 100% of the examined embryos, wherefore the findings shed new light on the role *Col4a2* plays in embryogenesis and tissue remodelling. In a translatory sense, it also enlarges the knowledge on the potential spectrum of defects associated with autosomal dominant porencephaly type 2 and might even be used as an argument for re-examining patients with high-resolution imaging techniques. However, the most important achievements are that our results stress the importance of DMDD for identifying mouse models for rare hereditary diseases and that they recommend the DMDD *Col4a2^em1(IMPC)Wtsi^* mutant strain as a model for systematically examining autosomal dominant porencephaly type 2. Unfortunately, the line is no longer actively bred and thus no longer at our disposal. According to the regulations of DMDD, the line was archived after the initial phenotype analysis but can be re-derived from the EMMA repository (https://www.infrafrontier.eu/search?keyword=EM:11598) for systematic phenotype examinations.

## MATERIALS AND METHODS

### Mouse line

*Col4a2^em1(IMPC)Wtsi^* C57BL/6NTac mice (*Mus musculus*) were generated at the Wellcome Sanger Institute (http://www.sanger.ac.uk/), by CRISPR/Cas9 technology leading to a CRISPR/Cas9-mediated deletion of critical exons to generate null mice. The resulting mutation is caused by deleting exon 18 (area deleted shaded in grey in following link: http://www.ensembl.org/Mus_musculus/Location/View?r=8:11312305-11449787;mr=8:11414602-11415015). As a consequence, a frame shift from phase 0 to 1 happened (http://www.ensembl.org/Mus_musculus/Transcript/Exons?db=core;g=ENSMUSG00000031503;mr=8:11414602-11415015;r=8:11413197-11417996;t=ENSMUST00000033899).

The resulting genotype was verified by end-point PCR screening and direct sequencing of the deletion amplicon.

#### Deleted sequence

[CCACATTGTAGAGCTGTTGCCATGGGGACAGTCCAGGTTTGATTGTGAGAAAACCCACACTATTAAACTAACTCTATATTCTGTGCACTTCTGGGCAATATACAAGGCTATTCTCACAGTGTTTTCTCTCCCCACCCCAGGGAGAACGGGGGGAACAAGGACCCCCAGGACCCTCTGTCTACTCGCCCCATCCATCCCTGGCAAAAGGTTGGTACAAACACTCCAGTGTATGACGTGGCCTCACATGGACCCTCTTCTGTCGGTAACAAAGTCATAAGCTCTCCAGACAAGTTCTTTAAGTGACTCAGAGACAGCCCAGCTTCCCTGGAGGTTTCAAAAGGAGTCACTAGAAATGGTCACTTTGGTTCTGGCTGACAACACTCAACTACCACCCAGGTGTGACCCTATCTGTGA].

#### Mutant allele (∼100 bp flanking sequence)

GCATCCATAGCCCCGGCAAGGGCTTCACGGGGAGATCCTCACGTGCTGTGGTGTTCCTTTTTATGTCTGTCTGGTGGTTAGGATGCAGGGACTCCCTGCTGGCAAGGGGAATGTCTTCATTTCTAGGGGATGCACAGGGTCTTGTTTTTAGCAGCCAGAATGCCACTGAGTCCGCTGAAATCACTGAAACCACTATGTGTCT.

### Gene expression analysis

Expression of *Col4a2* and *Col4a1* was analysed in homozygous (*n*=3), heterozygous (*n*=5) and WT littermate control (*n*=4) E9.5 embryos. The embryos were preserved in RNA later. Samples were run in triplicate using Life Technologies Taqman RNA to CT 1-Step Kit (part no. 4392938) using Life Technologies ViiA7 instrument.

The following Life Technologies assays were used: Col4a2 - Mm01216775_m1 FAM-MGB, Col4a1 - Mm01210125_m1 FAM-MGB and B2m_PL - Mm00437762_m1 VIC-MGB_PL (endogenous control).

For more information about this mouse line, see http://www.mousephenotype.org/data/genes/MGI:88455. This mouse strain can be ordered from the EMMA repository, with EMMA ID: EM:11598 (https://www.infrafrontier.eu/search?keyword=Col4a2).

As part of the DMDD program (https://dmdd.org.uk/) (Mohun et al., 2013; Weninger et al., 2014; Wilson et al., 2016), a total of four E14.5 *Col4a2^em1(IMPC)Wtsi^* homozygous mice (reference numbers in DMDD database, sex: DMDD6967, male; DMDD6968, female; DMDD6974, male; and DMDD6976, female) and three wild-type littermates (DMDD6966, male; DMDD6971, female; and DMDD6975, female) were processed for and imaged with the high resolution microscopy (HREM) method at the Francis Crick Institute, UK, according to a standardised procedure (Geyer et al., 2017a; Mohun and Weninger, 2012; Weninger et al., 2014; Wilson et al., 2016).

In brief, embryos were, after harvesting, screened under a dissection microscope and fixed in Bouin's for at least 24 h. Then they were rinsed in phosphate buffered saline (PBS), dehydrated in a graded series of methanol concentrations (10% increments with an additional step of 95% methanol, 2 h for each step), and infiltrated with methacrylate containing Eosin and Acridine Orange. They were then embedded in methacrylate resin (JB-4, Polysciences, Warrington, PA, USA) containing the same dyes and after polymerisation, the resin blocks were hardened at 90°C for 1–2 days. Using HREM, stacks of 3000–4000 inherently aligned digital images of near-histological quality were produced and converted to digital volume data with an isotropic voxel size of 2.55–3.0 µm^3^.

**Fig. 5.**
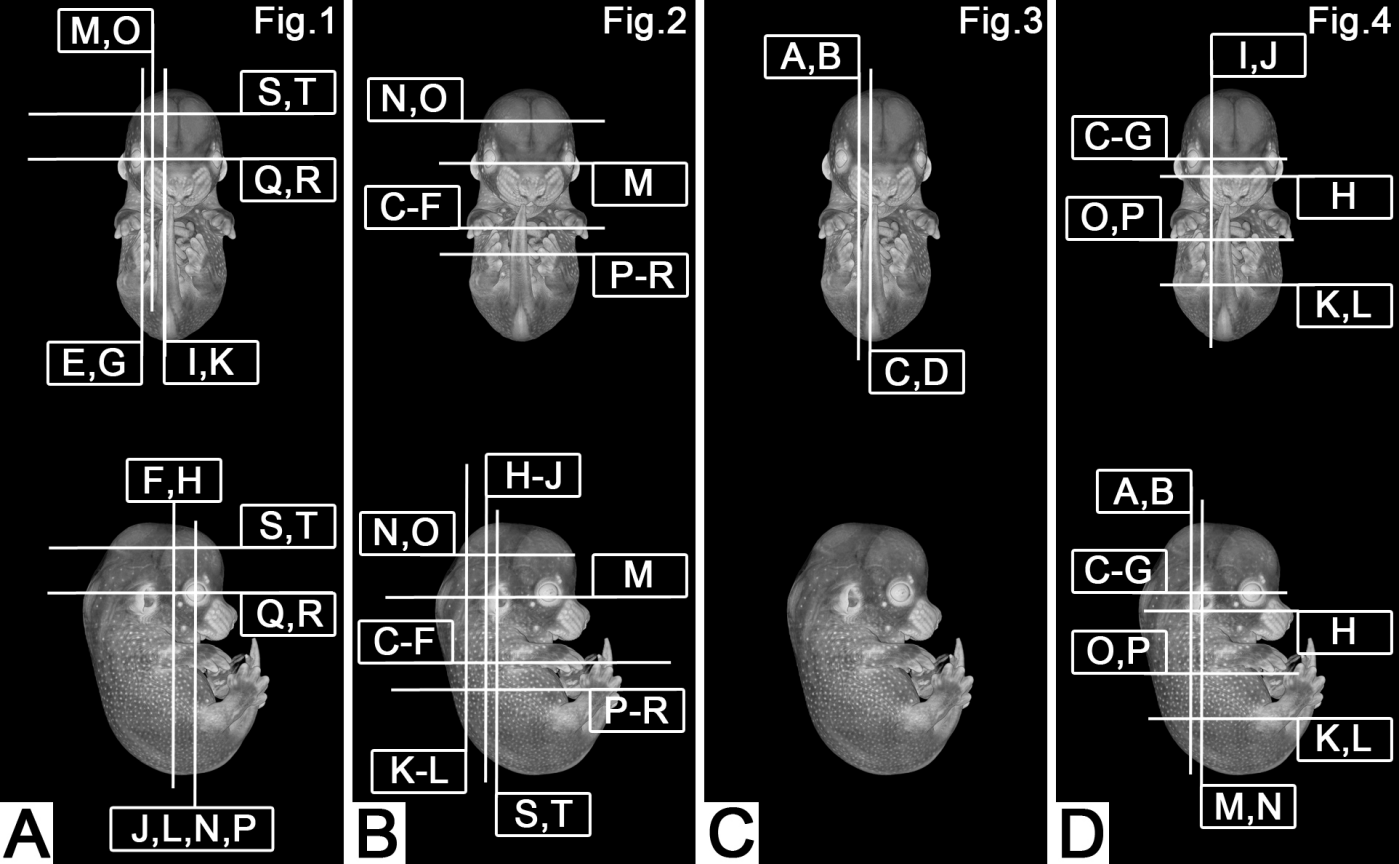
**Positions of virtual sections.** (A) Sectioning planes of images displayed in Fig. 1. Uppercase letters indicate figure panels. (B) Sectioning planes of images displayed in Fig. 2. Uppercase letters indicate figure panels. (C) Sectioning planes of images displayed in Fig. 3. Uppercase letters indicate figure panels. (D) Sectioning planes of images displayed in Fig. 4. Uppercase letters indicate figure panels.

The HREM data were analysed at the Medical University in Vienna, employing the display, volume rendering, and virtual dissection tools of the 64 bit OsiriX (http://www.osirix-viewer.com/) and Amira (6.1, https://www.fei.com) software packages. Phenotype abnormalities were scored according to standardised protocols (Weninger et al., 2014; Wilson et al., 2016). They were executed on volume-rendered virtual 3D models with voxel sizes of 3×3×3 µm^3^ and sequences of subsequent virtual, 3 µm thick section images cutting through the HREM volume data in various planes. For identifying abnormalities, we compared mutant data with the knowledge deduced from integrating information of data stemming from control embryos, which precisely matched the mutants in their developmental stages according to Geyer et al. (2017b). These reference data were created from systematic phenotype analysis of over 200 control embryos bred on the DMDD background and are already partly published (Geyer et al., 2017b; Geyer et al., 2017c). For documentation of abnormal morphology, we display images of sectioned volume models or 2D sections through the respective digital volume data of mutants and selected controls at section planes that are as closely comparable as possible (Fig. 5).

## Supplementary Material

Supplementary information
