# Supplementary material for: The Col4a2em1(IMPC)Wtsi mouse line: lessons from the Deciphering the Mechanisms of Developmental Disorders program
Source: Biol Open. 2019 Jul 22;8(8):bio042895. doi: 10.1242/bio.042895 (PMC6737985; doi:10.1242/bio.042895)
Supplement: Supplementary information [file biolopen-8-042895-s1.pdf]

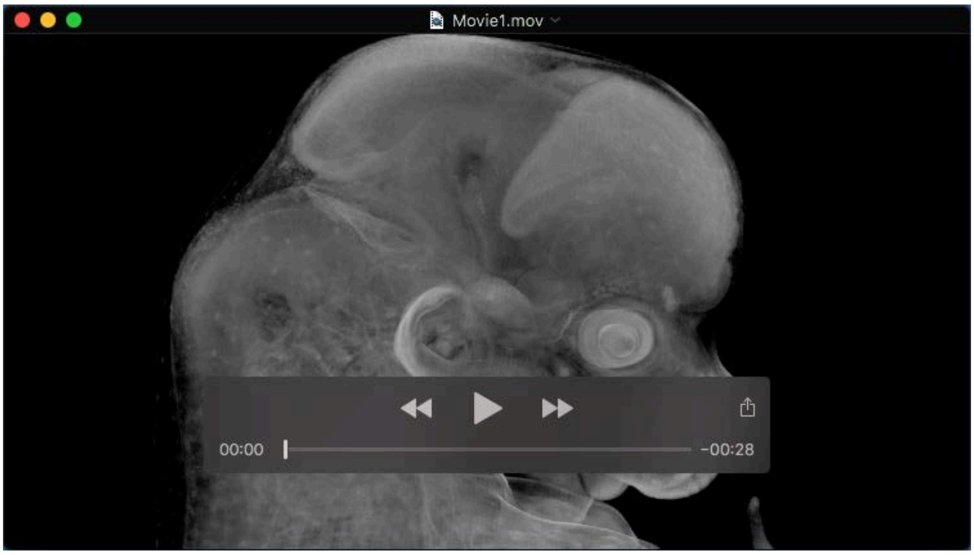

Movie 1 Defects of the nervous system in *Col4a2*$^{em1(IMPC)Wtsi}$ mutant mouse. Appearance of the forebrain in semitransparent volume model of head and neck, starting in lateral view. Note the irregular structure of the forebrain surface (for labelled malformations see Fig. 1).

Biology Open • Supplementary information

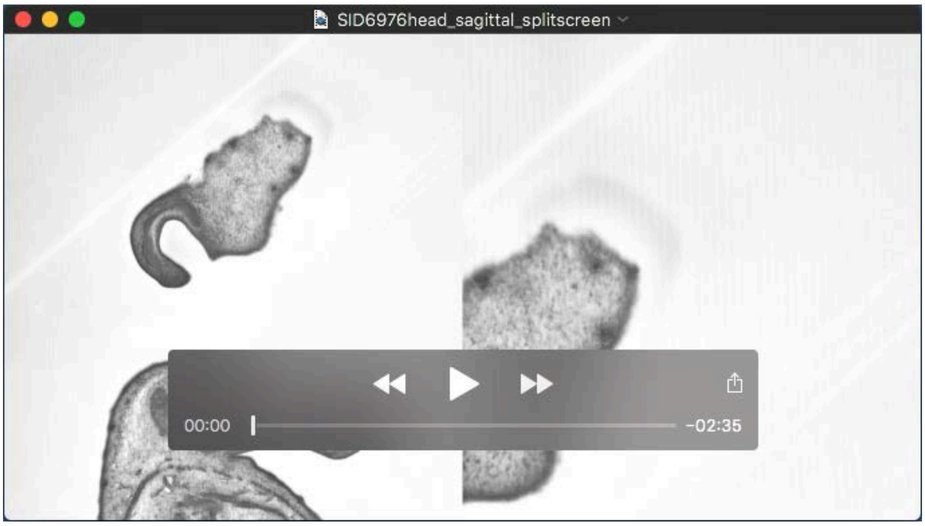

Movie 2 Defects of the nervous system and other organs in *Col4a2*$^{em1(IMPC)Wtsi}$ mutant mouse. Sagittal (ventral to the right) resections through HREM data. Note the following malformations: Irregular structure of the forebrain surface, tissue protrusions at the basal forebrain, cystic structures in the cortex of telencephalon, surface irregularities of the superolateral cortex of telencephalon, thickening of motoric portion of trigeminal nerve, haemorrhage in the trigeminal ganglion, abnormal oculomotor nerve (see Fig.1. for labelled malformations), retroesophageal right subclavian artery (see Fig. 2), fusion of vertebral arches (see Fig. 3), subepithelial cyst on hard palate, abnormal thyroid gland morphology, abnormal thymus topology (see Fig. 4).

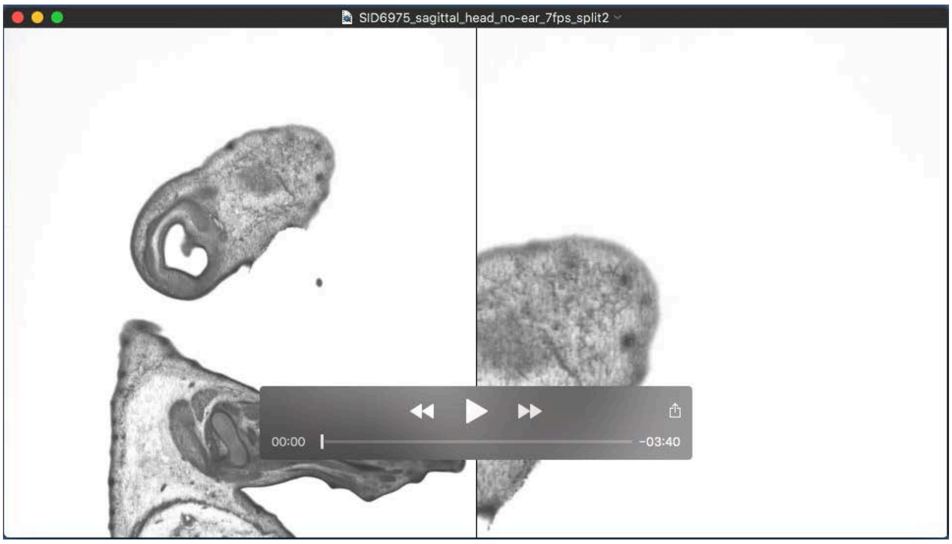

Movie 3 Normal nervous system in wildtype littermate control mouse. Sagittal (ventral to the right) resections through HREM data.

Table S1

Click here to Download Table S1

Biology Open • Supplementary information
